# Clinical Predictors of Delayed Discharges in Inpatient Mental Health Settings Across Ontario

**DOI:** 10.1007/s10488-018-0898-2

**Published:** 2018-10-03

**Authors:** Jerrica Little, John P. Hirdes, Christopher M. Perlman, Samantha B. Meyer

**Affiliations:** University of Waterloo, 200 University Ave West, Waterloo, ON N2L 3G1 Canada

**Keywords:** Alternate level of care, Delayed discharge, Inpatient mental health, Psychiatric hospital, InterRAI

## Abstract

Delayed discharges constitute an ongoing issue in psychiatric facilities. This study examined clinical predictors of 30-day delayed discharges in all designated inpatient mental health units within Ontario, Canada. Data for 76,184 inpatient episodes were obtained from 68 psychiatric facilities between 2011 and 2013. Risk factors for delayed discharges were analyzed using multivariate logistic regression. Indicators of functional, social, and cognitive impairment positively predicted delayed discharges, while symptoms of mental illness were inversely related. Policy makers and mental health care practitioners may utilize early predictors of delayed discharges to introduce treatment interventions and policies that reduce the risk of delays in mental health settings.

Delayed discharges from hospital occur when patients receive resources or services they no longer require while awaiting transfer to an alternate care setting (Canadian Institute for Health Information [CIHI] 2009). In Ontario, delayed discharges in inpatient mental health settings happen more frequently among older adults waiting for long-term care and individuals diagnosed with schizophrenia (Butterill et al. 2009; Little et al. 2015). Delays in discharge from hospital are associated with negative outcomes, such as accelerated declines in independence, social engagement, and resilience to illness and disease (Costa et al. 2012), as well as over-reliance on institutional settings (Glasby and Lester 2004). In addition to patients, hospital systems are also negatively affected by large volumes of delays. For instance, the restriction of available beds (Costa et al. 2012), avoidable monetary costs of ongoing treatment, and congested patient flow through facilities (Barnable et al. 2014) may occur as consequences of delayed discharges. As of December 2016, 10.5% of mental health beds in Ontario were occupied by patients waiting to be discharged (Cancer Care Ontario [CCO] 2017).

Risk factors for delayed discharges in inpatient mental health settings need to be understood to design interventions that reduce their duration, prevalence and incidence. However, identifying risk factors for delayed discharges has so far proven problematic. There are substantial differences in how delayed discharges have been defined; the cut-off points used for determining delays, as well as the assessment utilized, vary by study (Glasby and Lester 2004). Further, there is minimal research regarding delays in inpatient mental health settings specifically; greater emphasis is placed on length of stay, rather than delays. Finally, the lack of representative population data has posed statistical issues in being able to examine delayed discharge, a relatively uncommon outcome.

Diagnoses tend to be the most common characteristic associated with delayed discharge. Dementia has been correlated with delayed discharges in geriatric psychiatry (Hanif and Rathod 2008; Paton et al. 2004; Poole et al. 2014), although it remains poorly understood because it is often conflated with older age, which is also implicated in delays (Butterill et al. 2009; Kelly et al. 1998; Tanioka et al. 2013). Schizophrenia is another diagnosis that is frequently seen among those experiencing delays (Butterill et al. 2009; Kelly et al. 1998; Poole et al. 2014). However, diagnoses do not allow us to identify potential mechanisms related to delayed discharge. For instance, there is evidence to suggest that psychotic symptoms do not prevent successful discharges into the community (Leff and Trieman 2000; Ryu et al. 2006; Tanioka et al. 2013; Trieman and Leff 2002). Instead, factors such as disability and functional impairment (Butterill et al. 2009; Kelly et al. 1998; Koffman et al. 1996; Lewis and Glasby 2006; Paton et al. 2004; Poole et al. 2014; Tanioka et al. 2013), aggressive behaviour (Butterill et al. 2009; Park et al. 2009), social relationships and interpersonal dysfunction (Butterill et al. 2009; Paton et al. 2004; Poole et al. 2014; Springer and Paul 2008; Tanioka et al. 2013), and socioeconomic status (Butterill et al. 2009; Park et al. 2009) may pose a challenge for appropriate transition to community settings. These patterns suggest that delayed discharges are likely an indicator of poor health system response to the needs of certain patients.

There are significant gaps in the existing literature on risk factors for delayed discharges in inpatient mental health. For example, the literature suggests delayed discharges are a problem that affects patients and hospitals alike, yet little research has been conducted that examines prevalence estimates over time or the clinical needs of the population. Additionally, while several risk factors have been identified for delayed discharges, their replication in a large, externally valid sample is required. Considering the existing limitations of the literature to date, the purpose of this research is to identify risk factors at the time of admission to inpatient mental health units that predict future alternate level of care (ALC) designations, which is the administrative term that Ontario uses to denote delayed discharges. By identifying risk factors early on, health care providers will be able to pre-emptively manage such factors to reduce the possibility of an ALC designation. Drawing on existing literature, it was hypothesized that the following variables would increase the odds of being designated ALC: schizophrenia, dementia, older age, disabilities/impairments, aggression, interpersonal dysfunction, and low SES. Additionally, consultations with policy makers from the Ontario Hospital Association (OHA) were used to further explore potential risk factors that had not been established in the literature.

## Methods

### Study Design

A retrospective cohort study design was used to investigate predictors of delayed discharge. Independent variables were selected at the time of admission, and the number of delayed discharge days was retrieved from discharge assessments.

### Data

This study analyzed two data sources. Data from the Resident Assessment Instrument-Mental Health (RAI-MH) was used to describe characteristics of patients admitted to inpatient psychiatry. The RAI-MH data are contained in the Ontario Mental Health Reporting System (OMHRS), managed by the Canadian Institute for Health Information (CIHI). OMHRS was implemented provincially in 2005, when the Ministry of Health and Long-term Care mandated the use of the RAI-MH for all persons admitted to inpatient psychiatry in Ontario, Canada. Since its development, OMHRS has gathered RAI-MH assessments from 68 participating hospitals across Ontario.

The Wait Time Information System (WTIS) database, managed by Cancer Care Ontario (CCO), includes information related to ALC status. Several mental health units within the province adopted the WTIS system in 2011, with data available from all but two provincial psychiatric units up to the year 2013. Based on patient and episode ID, the number of ALC days were selected from WTIS and linked to the OMHRS dataset for the years 2011–2013. Due to facility delays in adopting the WTIS system, there were patient episodes in OMHRS that could not be matched; the loss of patient episodes was n = 8103.

### Study Sample and Setting

The sample included admissions to inpatient psychiatry beds in Ontario between March, 2011 and March, 2013. Short-stay patients, defined as those with a length of stay of three days or less, were excluded from the analysis because their episode was too short to have been at risk of delay. Forensic patients were removed because the factors that contribute to a delayed discharge could be due to legal or administrative reasons rather than clinical care needs. All other patients were retained in the sample (n = 76,184).

For statistical procedures, patient episodes were selected as the unit of analysis, allowing an individual with multiple episodes to be represented in the analysis multiple times. Episodes were selected because (a) they provide a better understanding of the prevalence of delayed discharges over time when individuals can be delayed multiple times and, (b) even when patient episodes are nested within the same person, each episode is an independent instance of a delay, and different clinical risk factors may have been involved. The number of episodes with a delayed discharge that occurred between 2011 and 2013 was n = 2074.

### Measurements

The RAI-MH is a comprehensive, standardized mental health assessment tool that is designed to appraise an individual’s needs, challenges, and strengths across a variety of domains, with the primary goal of assisting clinicians through person-centered assessment (Martin et al. 2009). The RAI-MH incorporates several different types of information into one tool, including demographic characteristics, clinical variables, scales, and Clinical Assessment Protocols (CAPs). Scales and CAPs, which are generated based on the scores assigned to relevant items embedded in the RAI-MH, are designed to alert clinicians to areas where an individual might be experiencing serious or imminent problems, i.e. risk of harm. The reliability and validity of the RAI-MH have been previously established in a variety of studies (Foebel et al. 2013; Gibbons et al. 2008; Hirdes et al. 2008; Jones et al. 2010; Martin et al. 2009).

#### Independent Variables

Based on the literature review and consultations with key stakeholders (e.g. members of the OHA), numerous variables from the RAI-MH were selected as independent variables of interest. Sociodemographic variables, such as age, gender, and living situation were included in the analysis, as well as variables related to social relationships (e.g. visit from a long-standing social relation) and service use (e.g. history of admissions to psychiatric units). Provisional Diagnostic and Statistical Manual Fourth Edition (DSM-IV) (APA 2000) diagnoses assigned by the attending psychiatric were also included, as well as the presence of an intellectual disability (referred to as dual diagnosis). Finally, a number of modifiable clinical factors were also examined. interRAI scales are calculated using responses to items contained in the RAI-MH, resulting in a continuous or ordinal measure of clinical status in a domain. Numerous clinical scales were included in this study, with higher scores representing greater symptom severity. The following scales were analyzed: Depressive Severity Index (DSI) (0–15), Positive Symptoms Scale—Long Version (PSSL) (0–24), Cognitive Performance Scale (CPS) (0–6), Aggressive Behaviour Scale (ABS), Activities of Daily Living Hierarchy (ADL-H) (0–16), Instrumental Activities of Daily Living (IADL) (0–30), and Social Withdrawal Scale (SWS) (0–6).

The DSI measures symptoms of depression, including sad, pained facial expression, negative statements, self-deprecation, guilt/shame, and hopelessness (Perlman et al. 2013). Similarly, the PSSL represents positive symptoms of psychosis, such as the presence of hallucinations, delusions, abnormal thought process, inflated self-worth, hyperarousal, pressured speech, and abnormal/unusual movements (Martin and Hirdes 2009). The CPS is an indicator of memory impairment, level of consciousness, and executive function (Martin and Hirdes 2009; Morris et al. 1994; Perlman et al. 2013). The ADL-H describes functional impairment in daily living and consists of personal hygiene, locomotion (walking/wheeling), toilet use, and eating (Martin and Hirdes 2009; Morris et al. 1999). The IADL measures functional capacity for complex daily living and includes meal preparation, managing finances, managing medications, transportation, and phone use (Martin and Hirdes 2009; Morris et al. 2013). The ABS describes an individual’s level of aggressive behaviour, encompassing verbal abuse, physical abuse, socially disruptive behaviour, and resistance of care (Martin and Hirdes 2009; Perlman and Hirdes 2008). Finally, the SWS is generated based on lack of motivation, reduced interaction, decreased energy, flat or blunted affect, anhedonia, and loss of interest (Rios and Perlman 2017).

CAPs are designed to provide a clinical summary of domains in the RAI-MH, with a focus on interpretation and evidence-based practice information. CAPs for inpatient psychiatry are provided in a manual (Hirdes et al. 2011). In the present study, two CAPs were of interest. The substance use CAP, which informs clinicians about an individual’s past and current history of substance abuse. This CAP includes triggers for current problematic substance abuse, and history of problematic substance abuse. The support systems for discharge (SSDIS) CAP alerts clinicians to an individual’s experiences of social isolation. This CAP includes triggers for reducing social isolation and family dysfunction, and improving close friendships and family functioning.

#### Dependent Variable

The dependent variable for this project was delayed discharges, measured using information on ALC collected from the WTIS database. WTIS measured ALC days as the total number of days a patient was designated ALC throughout their stay. For a patient to be designated as ALC, a physician must assess them according to the provincial guidelines set by CCO (2011). For the purpose of this study, the outcome was operationalized as a binary variable representing 30 + days of ALC. 30 days was chosen as the cut-off point because it represents a greater severity of delays, and it is consistent with how long-stays have been defined by CCO for ALC (CIHI, personal communication, October 1, 2014).

### Statistical Analysis

Analyses were performed using SAS software, version 9.4 of the SAS system for Windows. To determine which independent variables would be selected for the modeling process, a series of bivariate Chi square analyses were conducted. In addition to their theoretical relevance, variables that were significant at a corrected critical value were generally chosen for the modeling stage. If a variable contained more than one level or category, it was collapsed into one variable if no linear relationship existed (i.e. same risk status regardless of level). Otherwise, variables were separated into distinct categories with ‘0’ as the reference group.

To select variables for the multivariate model, the difference in column percentages, odds ratio (OR) values and statistical significance were all considered. Given the large sample size and the number of comparisons made (n = 80), a Bonferroni correction was applied to the p-value. The corrected p-value was α = 1 − (1–0.05)1/80 = 0.0006. Further, if a variable had a 95% CI overlapping 1.00, it was excluded from the model, unless there was a strong theoretical reason to leave it in. Variables with comparatively high column percentage differences and OR values were selected for evaluation in the multivariate modeling stage. For variables that contained a p value above 0.0001, but had only weak associations, the literature review was used to provide extra guidance for variable selection.

To determine the clinical predictors of delayed discharges at admission, a forward selection multivariate logistic regression modeling approach was adopted, with 30 + ALC days as the binary outcome. To begin, variables that were not expected to inter-correlate highly were entered into the model (e.g. sex, foreign language, and lifetime admissions to a psychiatric hospital). Following this step, variables were retained if they were significant, approaching significance, or if a theoretical reason existed to continue testing it. The c-statistic was also taken into consideration as variables were added and deleted. After refining the model, groups of variables that were known to inter-correlate were entered into the model sequentially (e.g. cognitive functioning and ADLs). As before, variables were retained based on significance values, contribution to the c-statistic, and/or guiding theory. The process was repeated until all variables had been tested, and only significant variables remained. Since the number of missing cases were small (2% of the total sample), they were deleted from the regression models.

## Results

### Descriptive Statistics

In the 30 + ALC days sub-sample in Table 1, there were a higher proportion of adults over the age of 44, men, individuals speaking a primary language other than English or French, and individuals with limited to no insight into their mental health. Individuals who experienced 30 + days of ALC were visited less often by a social relation, and were also less frequently married. Although marital status was not statistically significant, it was retained due to theoretical and clinical significance—the presence of a partner may translate into a resource for supporting discharge. Similarly, lifetime admissions to a psychiatric hospital was not statistically different between categories, but was tested for further modeling since it may act as a proxy for clinical complexity, severity, and persistence of mental illness. Additionally, several DSM-IV diagnostic categories were present more often in the 30 + ALC days sub-sample, such as disorders of childhood/adolescence, cognitive disorders, schizophrenia, and intellectual disabilities. Mood disorders and comorbid mental disorders occurred less frequently in the 30 + ALC days group.

**Table 1:** 30 + ALC designations by sample characteristics among mental health inpatients in Ontario, 2011–2013 (n = 76,184)

Variable	Variable level	% (N) of overall sample	30 + Days ALC	
			% (N)	OR (95% CI)
Age group	18–44	51.9 (38,839)	0.4 (153)	Reference
	45–64	35.7 (26,754)	1.1 (283)	2.70 (2.22–3.29)***
	65+	12.4 (9262)	5.0 (461)	13.24 (11.02–15.93)***
Gender	Female	50.9 (38,783)	0.9 (355)	Reference
	Male	49.1 (37,380)	1.5 (546)	1.61 (1.40–1.84)***
Primary language other than English/French	No	95.6 (72,827)	1.1 (811)	Reference
	Yes	4.4 (3357)	2.7 (90)	2.45 (1.96–3.05)***
Homeless	No	97.1 (74,009)	1.2 (873)	Reference
	Yes	2.9 (2175)	1.3 (28)	1.09 (0.75–1.60)
Insight into mental health	Full	20.4 (15,569)	0.2 (38)	Reference
	Limited	60.1 (45,775)	0.9 (406)	3.66 (2.62–5.10)***
	None	19.5 (14,840)	3.1 (457)	12.98 (9.32–18.09)***
Marital status	Married	23.3 (17,777)	1.16 (677)	Reference
	Unmarried	76.7 (58,407)	1.26 (224)	1.09 (0.94–1.27)
Visit by long-standing social relation/family member	< 3 days	67.3 (51,235)	0.8 (451)	Reference
	< 7 days	16.5 (12,572)	1.6 (136)	2.01 (1.66–2.44)***
	< 30 days	8.4 (6370)	2.1 (95)	2.78 (2.23–3.48)***
	30 + days	7.8 (5906)	4.5 (219)	5.96 (5.06–7.02)***
Number of lifetime psychiatric admissions	None	29.3 (22,325)	1.3 (293)	Reference
	1–3	36.6 (27,884)	1.0 (278)	0.76 (0.64–0.89)**
	4–5	13.8 (10,487)	1.1 (112)	0.81 (0.65–1.01)
	6+	20.3 (15,488)	1.4 (218)	1.07 (0.90–1.28)
Disorders of childhood/adolescence	No	97.8 (74,512)	1.1 (844)	Reference
	Yes	2.2 (1672)	3.4 (57)	3.08 (2.35–4.05)***
Delirium, dementia and amnestic and other cognitive disorders	No	94.4 (71,919)	0.6 (455)	Reference
	Yes	5.6 (4265)	10.5 (446)	18.34 (16.03–20.99)***
Schizophrenia and other psychotic disorders	No	64.9 (49,463)	1.1 (560)	Reference
	Yes	35.1 (26,721)	1.3 (341)	1.13 (0.99–1.30)
Mood disorders	No	47.6 (36,261)	1.9 (688)	Reference
	Yes	52.4 (39,923)	0.5 (213)	0.28 (0.24–0.32)***
Intellectual disability	No	96.2 (73,290)	1.1 (768)	Reference
	Yes	3.8 (2852)	4.7 (133)	4.62 (3.83–5.58)***
Comorbid disorders	No	59.1 (45,058)	1.3 (583)	Reference
	Yes	40.9 (31,126)	1.0 (318)	0.79 (0.69–0.90)**

In the 30 + days ALC column, % (N) indicates the column percentage of the frequency table, representing the percentage of individuals who were designated as 30 + ALC. Variations in sample size are due to the deletion of missing cases

*OR* bivariate odds ratio, *CI* confidence interval

*p < 0.05, **p < 0.01, ***p < 0.0001

In regards to clinical characteristics in Table 2, the IADL scale was associated with high rates of ALC status, with each increase in impairment leading to greater odds of being designated. Similarly, the ADL scale displayed a significant upwards trend for 30 + ALC designations. Other scales that demonstrated positive associations with 30 + ALC days included the CPS, ABS and PSSL. The DSI was the most negatively associated scale in relation to ALC status. Each increase in score on the DSI resulted in incrementally lower odds of being in the ALC group. Higher scores on the SWS also led to significantly lower odds of being in the 30 + ALC group. The 30 + ALC days sub-sample contained more individuals triggering the SSDIS CAP, and fewer individuals triggering the past and current substance abuse CAP.

**Table 2:** 30 + ALC designations by interRAI scales and CAPs among mental health inpatients in Ontario, 2011–2013 (n = 76,184)

Scale/clinical assessment protocol	Variable level	% (N) of overall sample	WTIS (30 + Days ALC)	
			% (N)	OR (95% CI)
Social withdrawal scale (SW)	0	23.4 (17,868)	1.4 (256)	Reference
	1–2	30.4 (23,180)	1.4 (312)	0.94 (0.80–1.11)
	3–6	46.12 (35,136)	1.0 (333)	0.66 (0.56–0.78)***
Positive symptoms scale—long (PSSL)	0	43.2 (32,905)	0.9 (297)	Reference
	1–3	20.4 (15,548)	1.4 (223)	1.60 (1.34–1.90)***
	4–8	22.1 (16,855)	1.5 (249)	1.65 (1.39–1.95)***
	9–24	14.3 (10,876)	1.2 (132)	1.35 (1.10–1.66)**
Instrumental activities of daily living scale (IADL)	0	60.4 (45,995)	0.2 (110)	Reference
	1–3	13.8 (10,514)	0.5 (50)	1.99 (1.43–2.79)***
	4–9	11.7 (8942)	1.3 (114)	5.39 (4.14–7.01)***
	10–18	7.4 (5671)	2.9 (167)	12.65 (9.93–16.12)***
	19–30	6.6 (5062)	9.1 (460)	41.69 (33.78–51.44)***
Activities of daily living scale (ADL)	0	86.7 (66,071)	0.5 (339)	Reference
	1–2	6.6 (5036)	3.4 (169)	6.73 (5.58–8.11)***
	3–4	2.7 (2051)	5.6 (115)	11.52 (9.28–14.30)***
	5–7	1.6 (1223)	7.9 (97)	16.70 (13.23–21.09)***
	8–16	2.4 (1803)	10 (181)	21.63 (17.94–26.08)***
Depressive Severity Index (DSI)	0	26.8 (20,458)	1.7 (362)	Reference
	1–3	32.3 (24,625)	1.4 (346)	0.79 (0.68–0.92)**
	4–7	24.7 (18,818)	0.8 (141)	0.42 (0.35–0.51)***
	8–15	16.1 (12,283)	0.4 (52)	0.24 (0.18–0.32)***
Cognitive Performance Scale (CPS)	0	65.1 (49,604)	0.3 (155)	Reference
	1–2	27.0 (20,601)	1.5 (308)	4.84 (3.99–5.88)***
	3–6	7.9 (5979)	7.3 (438)	25.22 (20.95–30.35)***
Aggressive Behaviour Scale (ABS)	0	73.8 (56,198)	0.7 (387)	Reference
	1–3	14.5 (11,017)	2.1 (226)	3.02 (2.56–3.56)***
	4–6	7.8 (5957)	2.8 (164)	4.08 (3.39–4.91)***
	7–12	3.9 (3012)	4.1 (124)	6.19 (5.04–7.61)***
Support systems for discharge (SSDIS) Cap	Not triggered	68.8 (52,438)	0.8 (438)	Reference
	Triggered	31.2 (23,746)	2.0 (463)	2.36 (2.07–2.69)***
Substance abuse CAP	Not triggered	54.4 (41,468)	1.8 (727)	Reference
	Triggered for past use	5.7 (4310)	1.4 (61)	0.81 (0.62–1.05)
	Triggered for current use	39.9 (30,406)	0.4 (113)	0.21 (0.17–0.26)***

In the 30 + Days ALC column, % (N) indicates the column percentage of the frequency table, representing the percentage of individuals who were designated as 30 + ALC. Variations in sample size are due to the deletion of missing cases

*OR* bivariate odds ratio, *CI* confidence interval

*p < 0.05, **p < 0.01, ***p < 0.0001

### Multivariate Logistic Regression

Table 3 displays the final multivariate logistic regression model for 30 + days of ALC.

**Table 3:** Multivariate logistic regression model predicting 30 + ALC days for mental health inpatients in Ontario, WTIS 2011–2013 (N = 74,732)

Variable	Group level	Parameter estimate (SE)	OR (95% CI)	χ^2^ p value
Age group	18–44 (ref)	–	–	–
	45–64	0.68 (0.12)	2.01 (1.62–2.49)	< 0.0001
	65+	1.03 (0.13)	2.89 (2.24–3.73)	< 0.0001
Gender	Female (ref)	–	–	–
	Male	0.36 (0.07)	1.42 (1.23–1.65)	< 0.0001
Primary language	English/French (ref)	–	–	–
	Other	0.34 (0.12)	1.40 (1.11–1.78)	0.006
Marital status	Unmarried (ref)	–	–	–
	Married	− 0.33 (0.09)	0.71 (0.59–0.85)	0.0002
Insight into mental health	Full (ref)	–	–	–
	Limited	0.64 (0.18)	1.89 (1.34–2.67)	0.0003
	None	0.65 (0.18)	1.92 (1.34–2.76)	0.0004
Lifetime admissions to a psychiatric hospital	0 (ref)	–	–	–
	1–3	0.16 (0.09)	1.18 (0.98–1.41)	0.08
	4–5	0.30 (0.13)	1.35 (1.06–1.73)	0.02
	6+	0.47 (0.11)	1.60 (1.29–2.00)	< 0.0001
Visit from a social relation	< 3 days (ref)	–	–	–
	< 7 days	0.11 (0.10)	1.12 (0.92–1.36)	0.3
	< 30 days	0.21 (0.12)	1.24 (0.98–1.56)	0.07
	30 + days	0.50 (0.10)	1.66 (1.36–2.01)	< 0.0001
Disorder of childhood/adolescence	No (ref)	–	–	–
	Yes	0.85 (0.18)	2.38 (1.69–3.36)	< 0.0001
Delirium, dementia and amnestic and other cognitive disorders	No (ref)	–	–	–
	Yes	1.14 (0.11)	3.11 (2.53–3.83)	< 0.0001
Mood disorders	No (ref)	–	–	–
	Yes	− 0.41 (0.09)	0.66 (0.56–0.79)	< 0.0001
Intellectual disability	No (ref)	–	–	–
	Yes	0.49 (0.12)	1.65 (1.30–2.10)	< 0.0001
Social withdrawal scale (SWS)	0 (ref)	–	–	–
	1–2	− 0.02	0.98 (0.81–1.17)	0.7
	3–6	− 0.19	0.83 (0.69–1.00)	0.05
Positive Symptoms Scale – Long (PSSL)	0 (ref)	–	–	–
	1–3	− 0.07 (0.10)	0.93 (0.77–1.13)	0.5
	4–8	− 0.07 (0.10)	0.93 (0.77–1.13)	0.5
	9–24	− 0.39 (0.12)	0.69 (0.54–0.87)	0.002
Cognitive Performance Scale (CPS)	0 (ref)	–	–	–
	1–2	0.51 (0.12)	1.61 (1.29–2.02)	< 0.0001
	3–6	0.69 (0.14)	1.90 (1.45–2.48)	< 0.0001
Instrumental Activities of Daily Living scale (IADL)	0 (ref)	–	–	–
	1–3	0.35 (0.18)	1.41 (0.98–1.94)	0.07
	4–9	0.93 (0.15)	2.35 (1.76–3.14)	< 0.0001
	10–18	1.29 (0.15)	3.21 (2.40–4.30)	< 0.0001
	19–30	1.45 (0.16)	3.81 (2.79–5.21)	< 0.0001
Activities of Daily Living scale (ADL)	0 (ref)	–	–	–
	1–16	0.44 (0.10)	1.55 (1.28–1.87)	< 0.0001
Depressive Severity Index (DSI)	0 (ref)	–	–	–
	1–3	− 0.14 (0.08)	0.87 (0.74–1.02)	0.09
	4–7	− 0.34 (0.11)	0.72 (0.58–0.89)	0.002
	8–15	− 0.57 (0.17)	0.57 (0.41–0.78)	0.0008
Aggressive Behaviour Scale (ABS)	0 (ref)	–	–	–
	1–12	0.16 (0.08)	1.17 (1.00–1.38)	0.05
Substance use CAP	Not triggered (ref)	–	–	–
	Triggered for past use	0.48 (0.15)	1.62 (1.22–2.15)	0.0008
	Triggered for current use	− 0.39 (0.11)	0.68 (0.54–0.85)	0.0006
Support systems for discharge (SSDIS) CAP	Not triggered (ref)	–	–	–
	Triggered	0.43 (0.07)	1.53 (1.33–1.77)	< 0.0001

*SE* standard error, *OR* odds ratio, *CI* confidence interval, *ref* reference group

$${C_{stat}}$$ 0.91

The predictor with one of the highest odds of 30 + ALC days was impairment on the IADL scale, which demonstrated an increase in odds for each sequential level of impairment, with those exhibiting the most impairment almost four times more likely to have 30 or more ALC days compared to those with no impairment. Other notable variables include moderate and severe cognitive impairment (OR 1.61 and OR 1.90, respectively) and middle and older age (OR 2.01 and OR 2.99, respectively), both of which also displayed increasing odds for each subsequent level. In terms of demographic characteristics, males and individuals who spoke a primary language other than English or French were both 1.4 times more likely to experience 30 + days of ALC. The clinical variables that showed positive odds of ALC status were limited to no insight into mental health (OR 1.89 and OR 1.92, respectively), disorders of childhood/adolescence (OR 2.38), disorders of cognition (OR 3.11), intellectual disabilities (OR 1.65), impairment in ADLs (OR 1.55), aggressive behaviours (OR 1.17), and a history of substance abuse (OR 1.62). Clinical variables that had lower odds of ALC status were severe positive symptoms of psychosis (OR 0.69), severe symptoms related to social withdrawal (OR 0.83), and moderate-to-severe symptoms of depression (OR 0.72 and OR 0.57, respectively). Variables indicating that an individual was socially isolated were positively predictive of ALC status, such as not being visited by a social relation in over a month (OR 1.66) and triggering the SSDIS CAP (OR 1.53), while being married was associated with a 29% decrease in the odds of ALC status. Finally, having six or more previous admissions to a psychiatric hospital had 1.6 times greater odds of 30 + ALC days.

## Discussion

The aim of the present research was to identify risk factors at the time of admission to inpatient mental health units that predict future delayed discharges. Through the logistic regression analysis of a large, representative sample, this study contributes novel information to the current literature. A unique finding is that variables representing mental health symptoms were negatively associated with long-stay delays, while characteristics related to cognitive, functional, and social well-being were positively associated. There are several ways to interpret the results. One explanation is that patients with severe symptoms of mental illness at admission may continue to present with clinical symptoms at discharge, and so they have lower odds of being perceived as a patient no longer in needs of services.

Another interpretation is that inpatient mental health services are primarily concerned with ameliorating symptoms of mental illness, and less involved in treating issues related to functional and social impairment. However, these characteristics are important for enabling successful transitions into the community (Ryu et al. 2006), and so if they are under-treated in inpatient mental health settings, they may contribute to delays. In such cases, a possible solution for reducing the number of delayed discharge days is to introduce interventions that focus specifically on cognitive, functional, and social impairment, such as group therapy and life skills management. Given that this is a new finding in the field of delayed discharges in inpatient mental health, further replication of the data using different samples is needed to support the validity of our interpretations.

While several variables were consistent with prior research on delayed discharges, there are notable exceptions. For instance, the finding that schizophrenia was not related to delays is contrary to the results of previous studies (Kelly et al. 1998; Butterill et al. 2009; Poole et al. 2014). One reason for the discrepancy could be differences in the methodology and statistical procedures that were used. For instance, it may be that after other variables have been accounted for, the effect of schizophrenia on delayed discharges is minimized. It is also possible that diagnoses of schizophrenia were historically related to delayed discharges, but in recent years, the relationship has weakened. This trend may be due in part to improved accessibility for various treatment and service options. In one Canadian health region, conformance to psychopharmacological treatments among those diagnosed with schizophrenia was fairly high, as well as some psychosocial interventions (Addington et al. 2012). The Canadian ‘At Home/Chez Soi’ project, which promotes stable housing interventions among homeless persons with serious mental illness, has also demonstrated efficacy in reducing hospital visits and other emergency health services (Mental Health Commission of Canada [MHOC] 2014). Altogether, the care needs of those diagnosed with psychosis may be met more often now than in previous years. Another possibility is that the population experiencing delayed discharges were primarily older adults with a diagnosis of cognitive disorders, and so the results were weighted more heavily to reflect this group. Due to the conflicting results surrounding schizophrenia and delays, it may be advisable to consider the presence of schizophrenia and other psychotic disorders when determining overall risk of a delay, but it should not be relied upon in the absence of more significant factors measuring clinical status directly.

Attempts to reduce delayed discharges must occur at two levels: the hospital and the community. At the level of the hospital, health care practitioners can act on information regarding risk factors for delays, although given the results of this study, it may require an extension in the scope of the services that are traditionally provided by mental health care practitioners. For instance, encouraging social relationships, managing aggressive behaviour, and improving symptoms of IADLs at the time of admission may lead to decreases in delayed discharges, even though these characteristics are not usually a focus of treatment. Additionally, incorporating risk factors for delayed discharges into discussions on early discharge planning would inform practitioners about the probability that a patient will be delayed, affording them extra time to plan for barriers to discharge and to mitigate the risks. In Ontario, information routinely collected through the RAI-MH could facilitate discussions about discharge, and the risks identified in this study could inform strategies to reduce the risk of delays.

Policy makers in the community can influence the services that are available for individuals with mental illness, but until this point, the types of resources that were needed for patients with delayed discharges were unknown. The identification of risk factors among the delayed discharge population provides insight into the services that patients require, enabling better care transitions. Mainly, community services that can accommodate patients with ADL, IADL, and cognitive impairment, as well as social isolation, would likely result in a decrease in rates of delays. Additionally, resources that specialize in caring for individuals with disorders of childhood/adolescence and dual diagnosis are also required, as they were identified as being at greater risk for delays as well. Policies can leverage these findings to support early discharge planning by encouraging care providers from both the hospital and the community to review delayed discharge risk and arrange necessary supports. Considering the average cost per day for a bed in one of Ontario’s four speciality psychiatric hospitals is $930 (Ministry of Health and Long-Term Care [MoHLTC] 2016), transitioning delayed discharges in hospital to outpatient services in the community would greatly diminish the costs associated with unnecessary ongoing inpatient treatment.

## Strengths and Limitations

One of the major strengths of this study is the inclusion of a representative sample of mental health inpatients in Ontario. By ensuring that almost all mental health inpatients across the province were included in the analysis, more accurate conclusions could be drawn from the results, as there was no subset of the population that was missing. Since delayed discharges rarely occur among mental health inpatients, it was imperative to obtain as many designated patients as possible, otherwise the statistical power to detect relationships may have been insufficient. Further, among the studies that did examine delayed discharges in mental health, only one performed a predictive regression analysis (Kelly et al. 1998), which is more useful than descriptive statistics in determining the risk and protective factors implicated in delays. Thus, this study has helped in advancing the field of research on delayed discharges in mental health settings by contributing a predictive, longitudinal analysis on delays.

There are some constraints to the RAI-MH and WTIS that are worth noting. Since the RAI-MH is a routine clinical assessment, the information collected is at the level of the individual, meaning that variables related to hospital and community services are unavailable. Delayed discharges represent a systemic issue extending beyond the person; in particular, geographic variations in available community services are likely influential predictors of delayed discharges. Future research needs to analyze data pertaining to hospitals and communities and link it to individual characteristics to obtain a full understanding of delays. Another weakness of the present study is that reliability of ALC designations could not be assessed. However, with the exception of schizophrenia diagnoses, the results of the present study were generally consistent with prior research on delayed discharges, indicating that the ALC designations were likely appropriate. While long-term cases were of primary interest to stakeholders involved in this project, restricting the analysis to 30 days of delays or more could also be considered a limitation. Examining all delayed discharges would increase the sample size of an already uncommon event, which could increase the accuracy of the regression results. Further, if there were differences in the regression results for any and 30 days of delayed discharges, intermediate groups could be created, refining our understanding of delays to a more intricate level.

Lastly, it can be considered both a strength and a limitation that admission episodes were used as the unit of analysis for this project. In terms of its advantages, using admission episodes to predict delays allows clinicians to identify risk and protective factors as soon as the patient begins their stay, which affords them as much time as possible to manage these factors before an ALC designation occurs. However, analyzing admission episodes alone can also be construed as a weakness, because the patient’s status on independent variables and level of risk may change throughout the course of their stay.

## Conclusion

In summary, a number of clinical and demographic characteristics were implicated in delayed discharges that occurred in mental health settings across Ontario, demonstrating that the needs of this this population are varied and complex. Policy makers and health care practitioners involved in mental health service delivery may benefit from the early identification of delayed discharge risk factors reported in this study, and may begin to design treatment interventions and policies that reduce the probability of delays, leading to reduced costs of treatment in hospital and improved care for patients. However, more work needs to be completed that addresses the reliability of ALC designations, as well as the environmental factors involved, as the availability of appropriate resources in the community is crucial for reducing delayed discharges across the province.
